# Validation of a Mobile Version of the American Shoulder and Elbow Surgeons Standardized Shoulder Assessment Form: An Observational Randomized Crossover Trial

**DOI:** 10.2196/16758

**Published:** 2020-07-17

**Authors:** Jingyi Hou, Qingyue Li, Menglei Yu, Fangqi Li, Yiyong Tang, Yi Long, Yamuhanmode Alike, Yuanhao Zhang, Maslah Idiris Ali, Congda Zhang, Weiping Li, Rui Yang

**Affiliations:** 1 Department of Orthopedics Sun Yat-sen Memorial Hospital Sun Yat-sen University Guangzhou China

**Keywords:** ASES, ePROM, smartphone

## Abstract

**Background:**

The American Shoulder and Elbow Surgeons Standardized Shoulder Assessment Form (ASES) questionnaire is an effective tool for evaluating shoulder joint function. The development and usage of a mobile version of the ASES questionnaire has the potential to save time, money, and effort.

**Objective:**

The aim of this study is to assess the equivalence between the paper and mobile versions of the ASES questionnaire and their acceptability among patients.

**Methods:**

The paper and mobile versions of the ASES questionnaire were used to evaluate the shoulder joint function of 50 patients with shoulder pain. This study included patients from the shoulder clinic of Sun Yat-sen Memorial Hospital. The intraclass correlation coefficient (ICC) and Bland-Altman method were used to evaluate the agreement (reliability) of the scores obtained by the two methods (paper versus mobile).

**Results:**

Of the 50 patients recruited from March 2018 to May 2019, 46 (92%) completed the study. There was a high agreement between the paper and mobile versions of the ASES questionnaire (ICC=0.979, 95% CI 0.943-0.987; *P*<.001). The mean difference between the scores of the mobile and paper versions was 1.0, and only 1/46 (2%) had a difference greater than the minimal clinically important difference of 12 points. About 75% of patients preferred the mobile version to the paper version.

**Conclusions:**

Our study shows that the mobile version of the ASES questionnaire is comparable to the paper version, and has a higher patient preference. This could prove to be a useful tool for epidemiological studies and patient follow-up over longer periods of time.

## Introduction

Shoulder pain is the third most common musculoskeletal problem and it can result in an inability to work and high medical costs [1]. It affects up to one-third of the general population, and is especially prevalent among the elderly [2]. When treating shoulder pain, we need to get information from the patients’ perspective to assess the severity of symptoms and the level of disability. The American Shoulder and Elbow Surgeons Standardized Shoulder Assessment Form (ASES) is a patient-reported outcome measure (PROM); it is widely used in sports medicine to evaluate shoulder function, and applied both in clinical research and clinical practice [3-8]. It includes two parts: the patient self-report section and the doctor's evaluation section. The patient self-report section has been validated in many languages and is considered to be a reliable and valid evaluation tool [3,6,7,9-14].

With the popularity of smartphones and the development of patient-focused software, the standard PROM is increasingly shifting from conventional paper and pen toward electronic administration of PROMs (ePROMs). Advantages of ePROMs include ease of use, reduced time of filling in the questionnaire, ease of data collection, high-quality data, reduced data attrition, reduced missing items, and improved patient compliance [15-19].

Some studies have compared the mobile and paper versions of PROM. Although most studies mention equivalence in scores, some studies have shown nonequivalence in scores [20], leading to the conclusion that simply digitizing existing PROMs without reliability testing cannot assure the reliability of ePROMs.

Our research group developed a mobile version of the ASES questionnaire. The aim of this study is to test the equivalence between the paper and mobile versions of the ASES questionnaire and their acceptability among patients.

## Methods

### Design

This was an observational randomized crossover trial. Participants completed both versions on the same day.

### Study Participants

Participants were chosen among patients of the shoulder clinic of Sun Yat-sen Memorial Hospital, Sun Yat-sen University. Inclusion criteria consisted of patients with shoulder pain, aged 18 years and above, and good written communication skills in Chinese. Exclusion criteria included limitations in understanding the Chinese language, difficulty in operating a touch screen device, a mental status that prevented the completion of the survey, or an unwillingness to participate. Ethical approval for this research was provided by the Ethics Committees of the Sun Yat-sen Memorial Hospital (SYSEC-KY-KS-2019-059).

### ASES Questionnaire

The self-report section of the ASES questionnaire is divided into two parts: (1) pain score and (2) daily activities. The total ASES score is derived from both parts. The pain score was obtained using the Visual Analogue Scale (VAS), which ranges from 0 (“No pain”) to 10 (“Worst pain”). For assessing the activities of daily living (ADL), 10 items are presented (Table 1) and graded on a 4-point ordinal scale. Scores ranged from 0 (“Unable to do”) to 3 (“Not difficult”). A weighted average was taken of the cumulative ADL score and the pain score, and this was merged into a total score. The formula is the following: ASES score = 5 × ([10 - ASES pain VAS] + ASES cumulative ADL score/3) [3].

**Table 1:** Patient self-evaluation: activities of daily living questionnaire^a^.

Activity	Right arm	Left arm
1. Put on a coat	0 1 2 3	0 1 2 3
2. Sleep on your painful or affected side	0 1 2 3	0 1 2 3
3. Wash back/do up bra in the back	0 1 2 3	0 1 2 3
4. Manage toileting	0 1 2 3	0 1 2 3
5. Comb hair	0 1 2 3	0 1 2 3
6. Reach a high shelf	0 1 2 3	0 1 2 3
7. Lift 10 pounds above shoulder	0 1 2 3	0 1 2 3
8. Throw a ball overhand	0 1 2 3	0 1 2 3
9. Do usual work. List:	0 1 2 3	0 1 2 3
10. Do usual sport. List:	0 1 2 3	0 1 2 3

^a^Patients circle the number in the box that indicates their ability to do the activity listed: 0=Unable to do; 1=Very difficult to do; 2=Somewhat difficult; 3=Not difficult.

### Mobile Version

**Figure 1:**
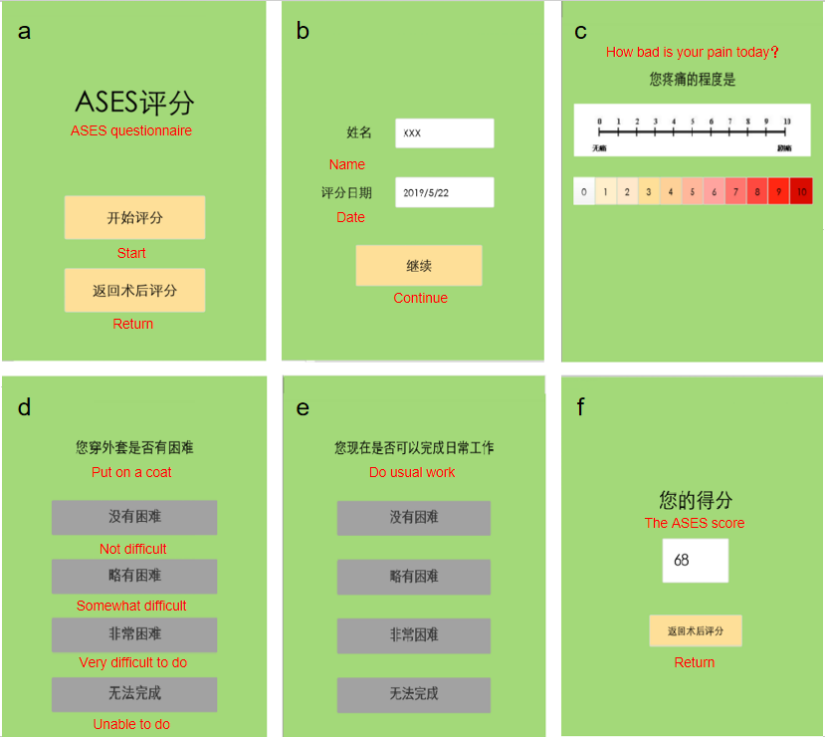
Screenshots of the iPad Mini screen showing (A) the login screen, (B) the home screen, (C) VAS scores, (D) and (E) ADL scores, and (F) the result screen. ADL: activities of daily living; ASES: American Shoulder and Elbow Surgeons Standardized Shoulder Assessment Form; VAS: Visual Analogue Scale.

The software of the mobile version of the ASES questionnaire was developed by our group. It can run on various operating systems including Android, iOS, and Windows. The major change to the mobile version of the ASES was that only one question was shown per page. Below each question, several options are placed and the screen changes to the next question when an option is selected. There are 11 options on a scale from 0 to 10 on the VAS page. As the patients may remember their answers to the first questionnaire and this could affect the results of the second questionnaire, the ADL items in the mobile version are set to appear randomly, with the options given in reverse order (ie, from 3 to 0). Once the patient clicked on an answer, the questionnaire automatically jumps to the next question, removing the possibility of unintentionally overlooking questions. For incorrectly filled questions, the mobile version allows the patient to go back and modify the answer. If the completion of the questionnaire is interrupted, the patient can retrieve it and continue without losing the previously entered information. Patients could log in to the software and fill out the questionnaire using an account and password given by the researchers. Once finished, the score would be displayed on the screen and stored on the server. Date and completion time are automatically recorded for each questionnaire. The score obtained using the paper version of the ASES questionnaire is input manually into the device. In this study, the test was administered on an iPad Mini (Apple Inc) with an A5 processor and a 20.1 cm screen (1024×768 screen resolution). Figure 1 depicts a screenshot of the mobile version of the ASES questionnaire.

### Study Procedures

With informed consent, patients were asked basic questions about demographics, smartphone familiarity, location, and clinical history; their answers were recorded by the researcher. Based on a computer-generated random number, patients either complete the mobile or the paper version of the questionnaire first. The aim of randomization is to prevent the sequence of trials from influencing the results. Patients were given the second questionnaire 2 hours after completing the first one. Each time a patient filled out the questionnaire, a stopwatch was used to measure the task completion time (accurate to seconds). Finally, the time required to complete the questionnaire was recorded. Patients were asked which questionnaire they preferred and the reason for their choice.

### Data Analysis

The same Excel sheet was used to summarize the data from the mobile and paper versions. Data from the mobile version could be automatically transferred to the Excel sheet with our software. For the paper version, two researchers would separately calculate each patient's score and enter it into the clinical data entry form established with EpiData software (EpiData Association). The data was then transferred to the same Excel sheet. As described above, a stopwatch was used to record the time required to sort the data. The data sorting time for the paper version is an average of the time taken by the two researchers. Descriptive statistics included the mean of aggregate scores, the SD, the mean score difference, and the SD of difference. Correlations between the scores obtained from the paper and mobile versions were assessed using the Pearson correlation coefficient, *r*. The intraclass correlation coefficient (ICC) and Bland-Altman method were used to evaluate the agreement (reliability) between the two methods (paper versus mobile) [21]. To determine whether the score differences between the two versions were clinically significant, we compared the difference with the minimal clinically important difference (MCID), which is the minimum change in score for a patient to notice differences in functional outcomes for the ASES questionnaire. As described in previous studies, 12 points was identified as the MCID for the ASES scores [22,23]. The *t* test was adopted for comparison of the time taken to fill out the form and sort data. Patient preference was studied by a simple content analysis.

## Results

### Participant Characteristics

From March 2018 to May 2019, 50 patients were enrolled in the study. Of these, 4 patients were excluded as they did not complete the second questionnaire. In total, 46 participants completed the study and were included in the final analysis. Details of the patients are shown in Table 2. The data of the patients in this study were consistent with the baseline population of our clinic, as shown in Multimedia Appendix 1.

**Table 2:** Details of patients included in the study.

Characteristics		Values
**Gender, n (%)**		
	Male	27 (59)
	Female	19 (41)
Age (years), mean (range)		43.87 (18-68)
**Diseases, n (%)**		
	Rotator cuff tear	20 (44)
	Frozen shoulder	6 (13)
	Impingement syndrome	3 (7)
	Instability of shoulder	5 (11)
	AC joint arthritis	5 (11)
	Superior labrum anterior and posterior (SLAP)	3 (7)
	Biceps tendonitis	4 (9)
**Prior use of smartphones, n (%)**		
	Yes	41 (89)
	No	5 (11)

### Consistency

The mean score of the paper version was 60.50 (SD 17.93) and the mean score of the mobile version was 61.46 (SD 18.17). The mean score difference was 0.96 (SD 0.24). The scores of the mobile version were strongly correlated with the scores of the paper version (Pearson *r*>0.98; *P*<.001).

**Figure 2:**
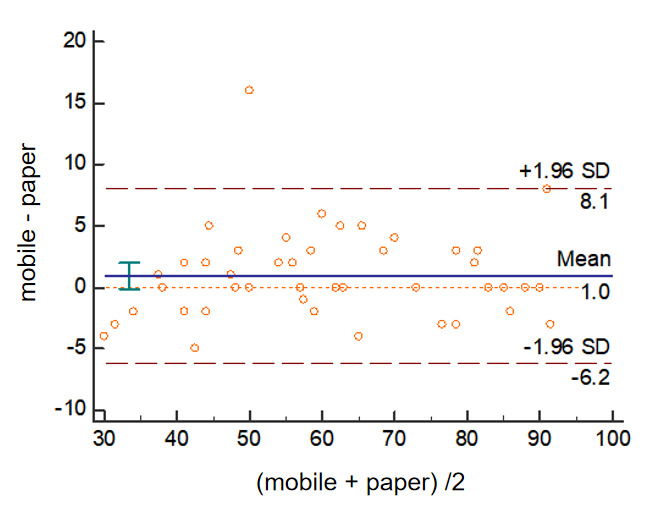
Bland-Altman plots between the paper and mobile versions of the ASES questionnaire. ASES: American Shoulder and Elbow Surgeons Standardized Shoulder Assessment Form.

As shown in Table 3, there was minimal variation between the mobile and paper versions. The ICC was 0.979 (95% CI 0.943-0.987; *P*<.001), confirming very good agreement between the versions. A Bland-Altman analysis for the ASES questionnaire showed that the mean difference between scores of the mobile and paper versions was 1.0 of a maximum of 100, and the 95% limits of agreement of the two methods was –6.2 to 8.1 (Figure 2). Only 1 patient of 46 (2%) had a score difference greater than the ASES MCID of 12 points (16 points), and the score difference was within 5 points for 93% (43/46) of cases. These results indicated excellent consistency between these two methods.

**Table 3:** Consistency analysis of paper and mobile versions.

Comparison	Intraclass correlation coefficient	*P* value	Bland-Altman analysis, mean difference
Paper versus mobile	0.979	<.001	1.0 (-6.2 to 8.1)

### Patient Acceptance

Patients were asked for their preference regarding the questionnaire version. Overall, 9 of 46 (19.6%) respondents expressed no preference as they found no obvious difference. Of those who expressed a preference, 28 of 37 (75.7%) preferred using the mobile version over the paper version, while 9 preferred the paper version. Among those who preferred the mobile version, 54% (15/28) found it easy to use and 29% (8/28) thought it was eco-friendly. Although 15.2% (7/46) of the patients had not used a smartphone or tablet before, more than half of that group (4/7) liked using the mobile version. Among patients who preferred the paper version, 8 of 9 thought the mobile version was not intuitive enough to use, as it only showed one question per page.

### Time

The paper version of the ASES questionnaire took an average of 1.76 minutes to fill in, while the mobile version took 1.01 minutes. The time taken for data sorting was 3.52 minutes for the paper version and 0.46 minutes for the mobile version (*P*<.001; Table 4).

**Table 4:** Time taken to fill out the form and sort data.

Group	Paper version	Mobile version	*P* value
Time taken to fill out the form	1.76 (0.48-3.35) minutes	1.01 (0.42-2.88) minutes	<.001
Time taken to sort data	3.52 minutes	0.46 minutes	<.001

## Discussion

PROMs have been widely used in sports medicine, both in clinical research and practice. Compared to the paper version of PROM, ePROM has many advantages. The objective of this study was to confirm the reliability of a mobile version of the ASES questionnaire and its acceptability among patients. The results of this study showed that there is a strong correlation in the ASES score between the mobile and paper versions. The high ICC of 0.979 indicates that the mobile and paper versions of the ASES questionnaire have excellent consistency. The results are in line with previous review articles comparing ePROM validation outcomes [24-28]. In most cases, the difference between the ASES scores of the mobile and paper versions was lower than 5 points. Very few cases (1/46, 2%) had a difference greater than the ASES MCID of 12 points, indicating that use of the ASES mobile version in place of the paper version would not affect the clinical interpretation of outcomes.

Overall, most participants preferred the mobile version of the ASES questionnaire, because it is eco-friendly and has a user-friendly interface. Even among patients lacking experience using smartphones, more than half preferred the mobile version. This preference may improve cooperation with PROMs.

The mobile version of the ASES questionnaire brings about many advantages. In clinical practice, several PROMs are typically administered simultaneously for shoulder pain [29-32]. A longer amount of time taken to fill in the PROMs may reduce the patient’s interest, which could lead to a drop in the quality of responses. In our study, the time taken to fill in the mobile version was significantly less than the paper version (1.01 versus 1.76 minutes; *P*<.001).

One major difference between the two versions is that the mobile version shows the next question only once the current question has been answered, ensuring that no items are missing from the testing process. This improved the reliability of outcomes. This also helps the patient focus only on one question without being distracted. The true benefit of the mobile version can be seen with data processing. The calculation process to obtain the total score of the ASES questionnaire is prone to error and not intuitive. Giving it an advantage over the paper version, the mobile version can automatically record test times, calculate total scores, and export the results to an Excel spreadsheet; it also provides an automated data retrieval system. This entire process is almost instantaneous. Conversely, for the paper version, it takes significantly more time to fill in the questionnaire and to process data. Our results show that handling the data takes 7 times longer in the paper version. The use of the mobile version is clinically significant as it can greatly reduce the workload of medical staff and save time, making clinical data collection easier. Considering the need to use several questionnaires during the same visit when evaluating shoulder function, the advantage of digitalization will be more pronounced. When sorting data for the paper version, the whole process was completed by two researchers separately to avoid calculation errors and transcription errors. We used the verification feature of EpiData to compare the two sets of data, and found no calculation or transcription errors.

Another advantage of the mobile version is that it can be used on mobile devices and be loaded on patients’ mobile phones directly, which means it can be completed at home and patients do not need to return to the hospital during long-term follow-up. In addition, the mobile version could automatically remind patients to take the test, thereby reducing dropout rates. In addition, patients can take the self-assessment test when they are feeling their worst to more accurately estimate the ASES score. This can help doctors develop better treatment or recovery plans.

We do acknowledge this study has some limitations. Due to the crossover design of this study, the impact of memory recall cannot be ignored. As the patient's pain score can change rapidly, the washout period could not be too long. In this study, the washout period was 2 hours. In addition, some researchers have tried new methods to overcome these challenges, such as creating two functionally equivalent halves of the item bank [33]. However, for the ASES questionnaire, there are relatively few items, and each item evaluates a specific functional direction, so this method cannot be adopted. Therefore, we explored a new approach. To further reduce the impact of memory recall, we set the ADL items to appear randomly in the mobile version, with the options given in reverse order from the paper version. Since these items are independent of each other and have no logical progression, such a change should be feasible in this situation. In the digitalization of other questionnaires, this method may be used as a reference when the questions are independent of each other. At the same time, the sequence by which the patients were given the questionnaire may affect the time taken to complete the questionnaire, although we found that among the first-filled questionnaires, the mobile version took less time than the paper version. Another limitation is that, since this software interface is in the Chinese language, it is currently only available to Chinese-literate countries and patients. Although we have verified the efficacy of this mobile version, further validation is still required upon the translation of this mobile version to different languages. Furthermore, people in some less-developed areas may not be able to use it due to a lack of advanced technologies.

ePROMs are becoming important in daily practice, and more of them will be used in clinical and research environments in the near future. The outcomes of our study show that the digitalization of the ASES questionnaire is feasible and useful. It reduces the workload of medical workers in collecting and processing data. Additionally, it saves patients’ time and allows them to evaluate their condition at any time. This can improve patient compliance and the accuracy of disease assessment, facilitating the implementation of personalized medicine. At the same time, as the difficulty of data collection is reduced, it will be beneficial for the development of real-world studies and predictive medicine.

In conclusion, our study shows that the mobile version of the ASES questionnaire is comparable to the paper version. More patients indicated a preference toward the mobile version of the ASES questionnaire as it is user-friendly and eco-friendly. The mobile version can save time for patients and doctors, and its automated data retrieval system allows for more efficient data collection and analysis. Therefore, this mobile version might prove to be useful in other epidemiological studies and long-term patient follow-up.

## Appendix

Multimedia Appendix 1 Details of the patients included in the study and all clinic patients in 2018.

## Abbreviations

ASES: American Shoulder and Elbow Surgeons Standardized Shoulder Assessment Form
ePROM: electronic patient-reported outcome measure
ICC: intraclass correlation coefficient
PROM: patient-reported outcome measure
MCID: minimal clinically important difference
VAS: Visual Analogue Scoring
