# Supplementary material for: Validation of a Mobile Version of the American Shoulder and Elbow Surgeons Standardized Shoulder Assessment Form: An Observational Randomized Crossover Trial
Source: JMIR Mhealth Uhealth. 2020 Jul 17;8(7):e16758. doi: 10.2196/16758 (PMC7395247; doi:10.2196/16758)
Supplement: Multimedia Appendix 1 [file mhealth_v8i7e16758_app1.docx]

**Table S1.** Details of patients included in the study and total patients in the clinic from 2018.

| Characteristics | | Included patients | | Total | | *P*value |
| --- | --- | --- | --- | --- | --- | --- |
|  |  | Number | Percentage | Number | Percentage |  |
| **Gender** | | | | | | |
|  | Male | 27 | 58.7% | 251 | 56.2% | ＞0.05 |
|  | Female | 19 | 41.3% | 196 | 43.8% |  |
|  | Mean age | 43.87 (18-68) |  | 48.69  (14-82) |  | ＞0.05 |
| **Diseases** | |  |  |  |  |  |
|  | Rotator cuff tear | 20 | 43.5% | 191 | 42.7% | ＞0.05 |
|  | Frozen shoulder | 6 | 13.0% | 63 | 14.1% |  |
|  | Impingement syndrome | 3 | 6.5% | 28 | 6.3% |  |
|  | Instability of Shoulder | 5 | 10.8% | 43 | 9.6% |  |
|  | AC joint Arthritis | 5 | 10.8% | 51 | 11.4% |  |
|  | SLAP lesion | 3 | 6.5% | 26 | 5.8% |  |
|  | Biceps tendonitis | 4 | 8.7% | 45 | 10.1% |  |

In 2018, we collected basic information on a total of 447 patients in the clinic. We compared it to patient data from this study. *T* test was adopted for comparison of the mean age of included patients and total patients. We used the chi-square test and Fisher exact test to compare the sex ratio and disease proportion. All *P* values were greater than .05. The results suggest that these 50 patients are representative as they have basic characteristics similar to those of the total patient population.
